# Advancing Diabetic Dental Restorations: A Comparative Analysis of Alveolar Bone Loss in Class II Composite Resin Versus Amalgam Fillings

**DOI:** 10.7759/cureus.72642

**Published:** 2024-10-29

**Authors:** Abdo Mohammed Mohammed Abdulrazzaq

**Affiliations:** 1 Department of Preventive Dental Science, Faculty of Dentistry, Najran University, Najran, SAU

**Keywords:** alveolar bone loss, amalgam fillings, composite resin, diabetic, restorative materials

## Abstract

Introduction

This study evaluated the impact of Class II composite resin and amalgam restorations on alveolar bone loss in diabetic patients, a population more susceptible to periodontal complications. The main objective was to determine whether the choice of restorative material impacts periodontal health, providing insights to optimize dental care for this high-risk group.

Materials and methods

This observational, comparative. cross-sectional study included 64 diabetic patients, divided into two groups based on their Class II restorations. Group 1 had 32 patients with composite resin restorations, while Group 2 comprised 32 patients with amalgam restorations. Both groups were matched for age and diabetes duration to ensure comparability. Periodontal health, specifically alveolar bone loss, was assessed through clinical and radiographic evaluations. The study analyzed the impact of the two materials on alveolar bone levels.

Results

Data from 64 diabetic patients (32 in Group 1 and 32 in Group 2) were statistically analyzed using PASW Statistics for Windows, Version 18.0 (Released 2009; SPSS Inc., Chicago, United States). Three statistical tests, descriptive statistics, two-sample t-test, and analysis of covariance (ANCOVA), were used. The results showed significant differences between the two groups, with composite resin restorations linked to greater alveolar bone loss.

Conclusion

The choice of restorative material significantly influences periodontal health in diabetic patients. Composite resin restorations were associated with a higher risk of alveolar bone loss and periodontal disease, emphasizing the need for careful material selection and regular periodontal monitoring in diabetic patients.

## Introduction

Periodontal disease, characterized by the destruction of the supporting structures of teeth, including the alveolar bone, is more prevalent and severe in patients with diabetes mellitus [1]. The bidirectional relationship between diabetes and periodontal disease has been widely recognized, with poor glycemic control being linked to increased risk and progression of periodontal disease [2]. Diabetic patients are also at an increased risk of associated alveolar bone loss [3]. The inflammatory response in diabetic patients is often more severe, leading to more pronounced bone destruction [4].

Composite resins and dental amalgams are the most used materials for Class II restorations. While dental amalgam has a long history of use due to its durability and cost-effectiveness, concerns about its mercury content have led to an increase in the use of composite resins [5]. There is a definite correlation between the effects of restoration on the periodontal health status [6]. Researchers found that patients with amalgam restorations had elevated plaque levels and gingival inflammation, suggesting amalgam may worsen periodontal conditions, particularly in individuals with predisposing factors like diabetes [7]. In this context, the choice of restorative material for dental restorations becomes crucial, as it may influence the progression of periodontal disease. This study aims to evaluate the impact of Class II composite resin versus amalgam restorations on alveolar bone loss in diabetic patients, hypothesizing that composite resins may lead to greater bone loss due to their potential to harbor more plaque and induce gingival inflammation.

## Materials and methods

This observational comparative cross-sectional study was conducted in the Faculty of Dentistry, Najran University, Saudi Arabia, from December 5, 2023, to August 24, 2024. The study was approved by the Najran University Research and Ethics Committee (approval No. 202409-076-023599 - 052877). Human examinations followed institutional and national ethical standards, adhering to the Helsinki Declaration (2013) and subsequent amendments.

Group allocation and sample size calculation

A total of 64 type 2 diabetics, aged 40-60 years were selected from selected from an initial pool of 489 diabetic patients who attended the specialized dental clinics at the Faculty of Dentistry, Najran University, Kingdom of Saudi Arabia, during the study period. The patients were divided into two groups of 32: Group 1 received class II composite resin restorations, and Group 2 received class II amalgam restorations. The service life of all restorations ranged from seven to 10 years. Clinical assessments included plaque index (PI), gingival index (GI), and radiographic evaluations of overhanging restorations, alongside measurements of alveolar bone loss using panoramic X-rays. Two clinicians conducted the assessments.

For the sample size calculation, we applied Crano and Brewer's formula [8] used in medical research, where $n = \frac{Nn*}{N + n*}$, the initial estimated sample size (n*) was calculated using the formula $n* = \frac{P (1 &minus; P)}{(SE) 2}$, P was assumed to be 0.5 to maximize the sample size, representing the estimated proportion of participants and SE, representing the standard error, was assumed to be 0.05.

Inclusion and exclusion criteria

The inclusion criteria for Group 1 were as follows: Diagnosed with diabetes for at least two years, with HbA1c levels of ≥7%, be aged between 40-60 years, possess at least 11 remaining teeth, and Class II composite resin restorations in place for 7-10 years. The inclusion criteria for Group 2 were as follows: Diagnosed with diabetes for at least two years, with HbA1c levels of ≥7%, aged between 40-60 years, have at least 11 remaining teeth, and Class II amalgam restorations in place for 7-10 years.

Exclusion criteria were the presence of systemic conditions other than diabetes, recent antibiotic or steroid use within two weeks prior to data collection, periodontal treatment in the past six months, edentulous elderly patients, and individuals with dental crowding or occlusal trauma.

Periodontal parameters examination

Two clinicians assessed plaque accumulation and gingival inflammation in each participant through a thorough oral clinical examination, evaluating periodontal tissue health. The clinical evaluation was based on the participants' current dental condition, with no prior dental procedures. The assessment focused on plaque index (PI) and gingival index (GI) using the two scoring systems proposed by Silness and Löe 1964 for PI [9] and Löe and Silness, 1963 for GI [10]. Each tooth surface (labial, lingual, mesial, distal) was inspected, excluding third molars, using William's periodontal probe to evaluate periodontal tissues.

Radiographic evaluation

Clinicians performed orthopantomography X-ray assessments to evaluate the alveolar bone loss and detect any irregularities or overhanging restorations that could indicate issues with dental work. A panoramic X-ray unit was used, with the resolution and height tailored to each patient's needs. The patient's chin was stabilized, and the occlusal plane was aligned horizontally, using a laser and touch screen for precise adjustments. Final volume positioning was fine-tuned as necessary. A computer-assisted system digitized and analyzed the radiographs, allowing for accurate linear measurements. These assessments were integrated into routine dental practice visits.

Alveolar bone loss measurements

Panoramic X-rays were used to evaluate alveolar bone loss in diabetic patients with Class II composite resin and amalgam restorations. Radiographs were deemed acceptable if key anatomical landmarks (cementoenamel junction (CEJ), alveolar bone crest (ABC), and apices (Ap)) were clear, without interference from restorations or image defects, and both proximal areas were measurable.

Calculation of the percentage of alveolar bone loss

The percentage of bone loss was calculated using the formula: $\frac{(CEJ-ABC) - 2mm)}{(CEJ-Ap) - 2mm)} &times; 100$, based on CEJ-ABC and CEJ-Ap measurements.

Statistical analysis

The analysis of the data incorporated descriptive statistics, a two-sample t-test, and an analysis of covariance (ANCOVA), which ensured that the study's findings were both statistically significant and clinically relevant. The use of ANCOVA was particularly important for controlling for covariates that could potentially confound the relationship between the type of restorative material and the periodontal health outcomes. This methodological approach enabled the derivation of reliable conclusions regarding the effects of composite resin and amalgam restorations on periodontal health in diabetic patients, thereby offering a stronger foundation for clinical recommendations. Statistical analysis was performed using PASW Statistics for Windows, Version 18.0 (Released 2009; SPSS Institute, Chicago, United States) to ensure comprehensive data processing and reliability. A p-value of less than 0.05 was considered statistically significant in all tests, providing a robust basis for interpreting the impact of restorative materials on periodontal health in diabetic patients and supporting stronger clinical recommendations.

## Results

The study involved a total of 64 diabetic patients, equally divided into two groups: Group 1 (n=32) received Class II composite resin restorations while Group 2 (n=32) received Class II amalgam restorations.

Descriptive statistics

The descriptive statistics offered a comprehensive overview of key periodontal variables, including mean values, standard deviations, and ranges for PI, GI, HbA1c levels, and alveolar bone loss percentage. This analysis facilitated clear comparisons between the two groups: composite resin restorations (Group 1) and amalgam restorations (Group 2). Group 1 exhibited higher median PI and GI values than Group 2 with similar interquartile ranges (Table 1), indicating greater periodontal inflammation, and experienced a higher bone loss percentage with a slightly broader range. Meanwhile, median HbA1c levels remained comparable across both groups with overlapping ranges. Group 1 had a higher median bone loss percentage compared to Group 2, with a slightly wider range.

**Table 1:** Comparison of descriptive statistics between the groups G1: Group 1 (composite resin restorations); G2; Group 2 (amalgam restorations); PI: plaque index; GI: gingival index; HbA1c: glycated hemoglobin

Statistics	Age (G1)	P.I (G1)	G.I (G1)	HbA1c (G1)	Bone Loss % (G1)	Age (G2)	P.I (G2)	G.I (G2)	HbA1c (G2)	Bone Loss % (G2)
Count	32	32	32	32	32	32	32	32	32	32
Mean	50.03	2.86	2.72	8.70	56.19	50.50	2.73	2.53	8.71	53.66
Standard Deviation	3.63	0.07	0.08	0.41	3.25	4.61	0.16	0.18	0.40	3.49
Min	45	2.70	2.50	8.10	52.00	45	2.25	2.20	8.10	45.00
25th Percentile	47.75	2.80	2.70	8.50	54.00	46.75	2.60	2.40	8.50	51.75
Median (50th Percentile)	49.00	2.90	2.75	8.60	55.50	49.00	2.75	2.50	8.60	53.00
75th Percentile	51.50	2.90	2.76	8.90	57.00	53.00	2.80	2.70	8.90	55.00
Max	59	3.00	2.90	9.40	65.00	60	3.00	2.80	9.40	60.00

T-test results

The findings indicate statistically significant differences between the two groups in PI (p-value<0.05), GI (p-value<0.05), and bone loss percentage (p-value<0.05), with no significant differences in HbA1c levels (Table 2). Initial t-tests were conducted to evaluate group differences in primary outcomes (PI, GI, and bone loss) without adjustment for confounding factors. These preliminary results revealed statistically significant differences, suggesting that restorative materials may influence periodontal health outcomes. However, while informative, the t-tests do not account for potential confounders such as age or HbA1c, limiting the ability to isolate the specific impact of restorative materials alone.

**Table 2:** Comparison of t-test results HbA1c: glycated hemoglobin

Parameters	t-Statistic	p-Value
Plaque Index	4.27	0.000069
Gingival Index	5.39	0.000001
HbA1c	-0.12	0.902457
Bone Loss (%)	3.00	0.003834

ANCOVA results

ANCOVA was to isolate the effects of restorative materials on periodontal health outcomes by controlling for covariates that could influence these outcomes independently. The results indicate that the treatment group significantly influences PI (p = 0.000081), GI (p = 0.000002), and bone loss percentages (p = 0.002394), while age is not a significant covariate for both PI and GI and marginally non-significant (p = 0.064246) for bone loss percentage (Table 3). On the other hand, HbA1c levels appear more affected by age than by the treatment group. In this study, both age and HbA1c levels were identified as crucial factors, as they can independently affect periodontal inflammation and bone loss. Controlling for these variables was essential to isolate the specific effects of the restorative material on periodontal outcomes.

**Table 3:** Comparison of ANCOVA results HbA1c: glycated hemoglobin; ANCOVA: analysis of covariance

Parameter	Source	Sum of Squares	df	F-Statistic	p-Value
Plaque Index	Group	0.268	1	17.84	0.000081
	Age	0.000007	1	0.000444	0.983256
Gingival Index	Group	0.582	1	28.25	0.000002
	Age	0.0067	1	0.324	0.571472
HbA1c	Group	0.00027	1	0.00184	0.965886
	Age	1.339	1	9.18	0.003581
Bone Loss (%)	Group	109.52	1	10.04	0.002394
	Age	38.74	1	3.55	0.064246

## Discussion

The findings of this study reveal that diabetic patients with Class II composite resin restorations exhibit significantly higher PI, GI, and alveolar bone loss percentages compared to those with amalgam restorations. Importantly, the lack of significant differences in HbA1c levels between the two groups suggests that the observed differences in periodontal health outcomes are more likely related to the type of restorative material rather than systemic glycemic control. Specifically, composite resins appear to contribute to increased periodontal risks in diabetic patients due to their surface roughness and potential for releasing chemical substances that provoke inflammatory responses.

The rough texture of composite resins enhances plaque accumulation, which in turn leads to higher levels of inflammation and bone loss. These results are consistent with earlier studies that have also associated restorations with higher periodontal complications. Some researchers found that Class II composite restorations had higher PI and GI scores (1.73, 1.58) compared to Class II amalgam restorations (1.57, 1.24) [11]. Additionally, a previously published study demonstrated that amalgam restorations contribute to alveolar bone loss in diabetic patients compared to non-diabetic individuals [7]. Another study validates the notion that subgingival restorations harm gingival and periodontal health, indicating that attachment loss occurs gradually and can be clinically observed within one to three years post placement. [12].

Overhanging margins in class II amalgam restorations heighten plaque buildup, worsening periodontal health and bone loss in long-term diabetic patients [13]. Restorations with overhanging margins can increase plaque accumulation and promote periodontal inflammation, facilitating a subgingival microflora linked to chronic periodontitis [14-16]. It can also lead to gingival inflammation, periodontal tissue destruction, and reduced alveolar bone height [15,16], increasing PI and GI and alveolar bone loss, thereby lowering periodontal scores in non-diabetics [16]. Previously reported data showed a wide prevalence range for overhanging Class II amalgam restorations, from 16.5% to 76% [17]. The current study builds on this by illustrating that composite resin restoration, while often preferred for its aesthetic benefits, may result in more severe periodontal damage in diabetic patients. This is largely due to the composite resin’s rough surface, which promotes plaque accumulation, leading to increased inflammation and subsequent bone loss. On the other hand, although amalgam restorations are smoother, they still present periodontal risks, such as gingival inflammation and plaque retention in diabetic patients.

Therefore, both materials pose risks to periodontal health, underlining the need for dental professionals to carefully consider the impact of different materials on plaque retention, inflammation, and bone loss and adopt stringent periodontal care protocols for diabetic patients. Although composite resins are favored for their aesthetic qualities, the findings suggest that their use in diabetic patients may exacerbate periodontal problems due to their rough surface. Conversely, while amalgam restorations are smoother, they still contribute to periodontal challenges. Thus, these results emphasize the need for a balance between aesthetic considerations and clinical outcomes in restorative dentistry for diabetic patients.

The implications for practice are clear: dental professionals must not only focus on the cosmetic aspects of restorations but also prioritize the long-term periodontal health of diabetic patients. Incorporating preventive strategies such as more frequent periodontal cleanings, enhanced home-care instructions, and regular monitoring of periodontal health can help mitigate these risks.

Limitations

Although this study offers valuable insights, it is essential to acknowledge several limitations. The sample size of 64 participants restricts the generalizability of the findings; a larger cohort would improve external validity. Furthermore, the cross-sectional design captures only a momentary view of periodontal health, limiting understanding of changes over time. The absence of data on smoking and obesity status, both known to affect periodontal outcomes, is another limitation. Additionally, factors related to the quality of dental restorations, such as the smoothness of Class II restorations and proximal overhang sizes, may have influenced disease progression. Future studies should address these aspects for a more thorough understanding.

Recommendations

The findings of this study suggest numerous opportunities for future research. Investigating innovative restorative materials that combine the aesthetic qualities of composite resins with the smooth surface characteristics of amalgam is essential. Additionally, fostering collaboration between dental and endocrinological specialists will enhance understanding of the complex interactions between diabetes management and the effect of restorative materials on periodontal health. Exploring how variations in glycemic control influence the relationship between these materials and periodontal tissues could provide valuable insights for developing customized treatment strategies tailored to the needs of diabetic patients.

## Conclusions

This study provided significant evidence that Class II composite restorations were associated with greater alveolar bone loss, PI, and GI compared to amalgam restorations in diabetic patients. The results suggested that the choice of restorative material plays a crucial role in the periodontal health of diabetic patients, with composite resins potentially exacerbating periodontal deterioration. Clinicians should consider these findings when selecting restorative materials for diabetic patients, balancing the benefits of aesthetic outcomes with the need to minimize periodontal risk. Additionally, the study highlighted the importance of regular periodontal monitoring and proactive management in diabetic patients, particularly those with composite resin restorations, to prevent accelerated periodontal disease progression. These insights contribute to a better understanding of the impact of restorative materials on periodontal health in a vulnerable population and guide future clinical practice.
